# Anti-obesity and Hypolipidemic effects of garlic oil and onion oil in rats fed a high-fat diet

**DOI:** 10.1186/s12986-018-0275-x

**Published:** 2018-06-20

**Authors:** Chao Yang, Lihua Li, Ligang Yang, Hui Lǚ, Shaokang Wang, Guiju Sun

**Affiliations:** 1 Key Laboratory of Environmental Medicine and Engineering of Ministry of Education, and Department of Nutrition and Food Hygiene, School of Public Health, Southeast University, No. 87 Ding Jia Qiao Road, Nanjing, 210009 China; 2 Second Clinical Medical College, Nanjing University of Traditional Chinese Medicine, Nanjing, 210046 China

**Keywords:** Garlic oil, Onion oil, Anti-obesity, Hyperlipidemia, Rats

## Abstract

**Background:**

Until now, little research concerning the lipid-lowering and anti-obesity functions of garlic oil and onion oil has been performed. The objective of this study was to explore the effects of garlic oil and onion oil on serum lipid levels in hyperlipidemia model rats, to provide a scientific basis for the prevention of hyperlipidemia through a dietary approach, and to explore the potential health benefits of garlic and onion.

**Method:**

Ninety-six male Sprague-Dawley rats were randomly allocated into eight groups based on their body weight and serum levels of triglycerides (TG) and total cholesterol (TC). The rats received repeated oral administration of volatile oils extracted from garlic and onion for 60 days. Serum lipids and parameters of obesity were examined.

**Results:**

The volatile oils suppressed the HFD-induced body weight gain and tended to decrease adipose tissue weight. The oils decreased the levels of TG, TC and LDL-C and increased the serum level of HDL-C compared with the rats in the hyperlipidemia model groups (*P* < 0.05). The oils were also effective at improving the lipid profile and alleviating hepatic steatosis.

**Conclusion:**

Our results implied that garlic oil and onion oil have anti-obesity properties that can counteract the effects of an HFD on body weight, adipose tissue weight, and serum lipid profiles.

**Electronic supplementary material:**

The online version of this article (10.1186/s12986-018-0275-x) contains supplementary material, which is available to authorized users.

## Background

Garlic (*Allium sativum* L.) has been widely used as a foodstuff and, for many centuries, has also been used as a traditional medicine due to its perceived effects in preventing and curing ailments [1]. China has 33.3 million square meters devoted to cultivating garlic and produces more than 500 million tons of garlic, accounting for ½ of the total area of cultivated garlic in Asia and 1/3 of the total area of cultivated garlic in the world [2]. Several organosulfur compounds present in garlic oil have been shown to possess numerous biological activities. The four most important organosulfur compounds, considered to be the major biological agents, are diallyl sulfide (DAS), diallyl disulfide (DADS), diallyl trisulfide (DATS) and allylmethyl trisulfide [3]. A series of biological benefits, such as hypolipidemic and hypocholesterolemic effects [4], antioxidant potential [5, 6], and antimicrobial activity [7], have been reported. The pungent fractions of garlic are mostly sulfur-containing moieties, while its two chemical groups, namely, flavonoids and ALK (EN)-based cysteine sulfoxides (ACSOs), have marked effects on human health [8].

Onion (*Allium cepa* Linn.) is also one of the oldest cultivated plants, with its cultivation dating back to before 5000 BC. World onion production has increased by at least 25% over the past 10 years to the current output of approximately 44 million tons, making it the second most important horticultural crop after tomatoes [8]. Onion is rich in sulfur-containing compounds and is used as a foodstuff, a flavoring, a condiment and folk medicine. According to traditional Chinese medicine, onions’ special features are warm and hot tastes, together with functions such as warming the lungs to reduce phlegm, warming the stomach during digestion, detoxifying and destroying intestinal worms, decreasing swelling and soreness, and decreasing blood pressure and blood lipids. As a result, onions are used for expelling worms in children, curing rheumatoid arthritis, treating the swelling and pain associated with athlete’s foot or dermal ulcers, dysentery, pertussis, insomnia and other conditions [9, 10]. Onions contain a considerable number of compounds that are highly beneficial for human health and that have been reported to have anti-hyperglycemic and anti-hyperlipidemic effects on diabetic rats [11]. ACSOs are also some of the active components in onion oil. The main components of onion oil are sulpho compounds, including propane disulfide, disulfide propylene, S-methyl cysteine, and others [12].

Patients with dyslipidemia are at increased risk for cardiovascular disease (CVD), which is the main cause of premature death and has been a major cause of disability and ill health in recent years [13, 14]. In China, the situation is very serious, and the number of patients with dyslipidemia has reached 160 million, with annual numbers of disability and death originating from dyslipidemia-associated diabetes, stroke, cerebral infarction, hemiplegia, or myocardial infarction also increasing [15]. As a result, it is important to find reasonable and effective ways to prevent, control, and improve dyslipidemia to provide significant reductions in atherosclerosis and cardio-cerebral vascular diseases. Obesity is one of the most prevalent heath conditions that may foster various diseases, such as dyslipidemia, metabolic syndrome, hypertension and increased risk of cardiovascular mortality [16]. There have been few studies concerning the lipid-lowering and anti-obesity properties of garlic oil and onion oil prior to the current study. The objectives of this study were to explore the influence of garlic oil and onion oil on serum lipid levels in dyslipidemia model rats, to provide a scientific basis for the prevention of dyslipidemia through a dietary approach, and to explore the potential health benefits of garlic and onion.

## Methods

### Chemicals

Chemicals such as casein, cholesterol, bile salts, DL-methionine, choline chloride and custard powder were bought from Sinopharm Chemical Reagent Company(Shanghai, China), while the vitamin mixture was purchased from Days Restoration Exploration Food Co. Ltd. in Beijing. The lard, cornstarch, sucrose and canola were purchased in the market.

### Preparation of garlic oil and onion oil

Garlic oil and onion oil from the fresh bulbs of *Allium sativum* and *Allium cepa,* respectively, were prepared by steam distillation using an essential oil extractor (provided by Nanjing WanQing Chemical Glassware Instrument Co. Ltd) and an electric heater thermostat (made by Tongzhou Shen Tong Heater Co. Ltd) with the existing technology in our laboratory [17]. For the garlic oil, fresh garlic cloves were chopped, soaked in 3.5-fold distilled water at 35 °C for four hours, and then subjected to hydrodistillation in essential oil testing equipment for 2 hours. To attain the onion oil, fresh bulbs were chopped, soaked in 1.5-fold distilled water at 35 °C for 2.5 h, and then subjected to hydrodistillation in essential oil testing equipment for 2.6 h. All of the essential oils obtained were dehydrated with anhydrous sodium sulfate and stored in the dark at room temperature until use. The chemical composition of the garlic and onion oil preparations were determined by gas chromatography-mass spectrometry (GC-MS). DADS and DATS accounted for 64.7% of the composition of the garlic oil, while sulfur nitrogen compounds made up 80% of the composition of the onion oil. **These results were obtained in our previous studies** [18] **and are similar to those results found in the study by Zhao et al.** [19].

### Induction of the hyperlipidemic and obese rat model and experimental design

Ninety-six male Sprague-Dawley rats weighing 120~ 150 g were obtained from the Experimental Animal Center in Zhejiang, and the license number was SCXK (ZHE) 6008–0033. They were housed six per cage in a room with a 12/12-h light/dark cycle and an ambient temperature of 22 ~ 25°C. All rats were fed a pelletized commercial chow for 7 days after arrival. Hyperlipidemia was induced in the rats by feeding them a high-fat diet based on the AIN-93 semisynthetic diet; the detailed ingredients of the diet are shown in Table 1. The obesity model was judged to be successful when the body weight in the intervention groups was 10% higher than that in the control group [20]. The mineral mix and vitamin mix were followed the recommended doses in AIN-93 M [21].

**Table 1:** Composition of the experimental diets (%)

Ingredient	Basal diet	High-fat diet
Casein	23	20
Corn starch	32	29
Sucrose	31	25.3
Fiber	4	4
Canola	5	0
Lard	0	10
Custard powder	0	5
Cholesterol	0	1.5
Bile salt	0	0. 2
Mineral mix	3.5	3.5
Vitamin mix	1	1
DL- Methionine	0.3	0.3
Choline chloride	0. 2	0. 2
Total	100	100

According to the body weight and serum levels of triglycerides (TG) and total cholesterol (TC), ninety-six male rats were randomly allocated into eight groups, including the normal control group (NC); the hyperlipidemia model group given water (HFD-A); the hyperlipidemia model group given Tween 80 (HFD-B); the low, moderate and high dose garlic oil groups (GO-L, GO-M, and GO-H, treated with 11.6 mg/kg·bw/d, 46.3 mg/kg·bw/d, and 92.6 mg/kg·bw/d, respectively); and the low and high dose onion oil groups (OO-L and OO-H, treated with 46.3 mg/kg·bw/d and 92.6 mg/kg·bw/d, respectively).

The garlic oil and onion oil were dissolved in 1% Tween 80 [22] and administered to the rats every day by intragastric administration for 60 days. The HFD-A group and the NC group were prepared to evaluate the success of the hyperlipidemia model, while the HFD-B group was used to eliminate the effects of tween-80 on the rats and to demonstrate that Tween 80 had no effect on the hyperlipidemic rats.

All investigations were carried out in accordance with the ‘Principles of Laboratory Animal Care’ by the NIH and the protocol for animal study of the Animal Management Committee of Jiangsu Province, China.

### Collection of blood and tissue samples

At the end of the experiment, all rats were anesthetized with pentobarbital sodium and sacrificed by cervical decapitation after overnight fasting for 12 h. Blood samples were collected from the femoral artery; the liver, kidneys, and adipose tissue (including the epididymal fat pads and perirenal fat pads) were surgically removed and weighed immediately. The blood samples were allowed to coagulate and were then centrifuged at 3000 *r/min* for 15 minutes. The serum was stored at -20 °C until analysis. The liver, kidney and adipose tissues were weighed, immediately placed on ice bags, and then stored at − 80 °C for further analysis. The naso-anal length (distance from nose to anus) of the animals was also measured on the 60th day before the animals were sacrificed. The animal experimental protocols were conducted according to guidelines of the Institutional Animal Care and Use Committee of Southeast University.

### Analysis of lipid concentrations in serum

Levels of TG, TC, high-density lipoprotein cholesterol (HDL-C) and low-density lipoprotein cholesterol (LDL-C) were enzymatically analyzed using a commercial kit (Nanjing Jiancheng Bioengineering Institute, Jiangsu, People’s Republic of China).

*Calculation of some other evaluation parameters* [23–25]

$$ \mathrm{Lee}\ \mathrm{index}=\frac{\sqrt[3]{\mathrm{body}\ \mathrm{weight}\ \left(\mathrm{g}\right)}\times {10}^3}{\mathrm{naso}\hbox{-} \mathrm{anal}\ \mathrm{length}\ \left(\mathrm{cm}\right)} $$

$$ \mathrm{The}\ \mathrm{ratio}\ \mathrm{of}\ \mathrm{fat}\ \mathrm{to}\ \mathrm{body}\ \mathrm{weight}=\frac{\mathrm{epididymal}\ \mathrm{fat}\ \mathrm{pads}+\mathrm{perirenal}\ \mathrm{fat}\ \mathrm{pads}}{\mathrm{body}\ \mathrm{weight}}\times 100\% $$

$$ \mathrm{Atherosclerosis}\ \mathrm{index}=\frac{\mathrm{TC}-\mathrm{HDL}-\mathrm{C}}{\mathrm{LDL}-\mathrm{C}} $$

### Statistical analysis

Data were reported as the mean ± SEM ($\overline{x}\pm s$). All statistical analyses were performed using SPSS 17.0 software. The significances of differences among the groups were analyzed by one-way analysis of variance (one-way ANOVA), and differences were separated using Tukey’s multiple comparisons test. Values of *P* < 0.05 were considered statistically significant.

## Results

### Body weight and food intake

As shown in Table 2 and Additional file 1: Table S1, the initial body weights of the eight groups were not significantly different. However, after 60 days of treatment, the final body weights and body weight gains were significantly lower in the NC group. Additionally, the final body weights and body weight gains in the HFD-A and HFD-B groups were also considerably higher than those of the rats treated with garlic oil and onion oil, although they had received the same diet and had similar food intake. Additionally, garlic oil and onion oil had no effect on the food intake of the rats fed the high-fat diet, as shown in Table 2 and Additional file 2: Table S2.

**Table 2:** Body weight and food intake of rats fed experimental diets for 60 days ($\overline{x}\pm s$, *n* = 12)

Group	Initial body weight (g)	Final body weight (g)	Body weight gain (g/d)	Food intake (g/d)
NC	166.8 ± 23.5	449.9 ± 38.7	6.3 ± 0.8	27. 4 ± 6.1
HFD-A	170.9 ± 26.7	540.3 ± 26.7^a^	8. 2 ± 0.8^a^	24. 4 ± 2.9^a^
HFD-B	166.8 ± 21. 2	535.7 ± 40.7^a^	8. 2 ± 0.8^a^	23.6 ± 3.0^a^
GO-L	159.6 ± 16.8	469.1 ± 37.8	6.8 ± 0.9^b^	22.8 ± 1.6^a^
GO-M	151.7 ± 18.6	447.9 ± 23.8^b^	6.6 ± 0. 4^b^	23.8 ± 4.0^a^
GO-H	157.1 ± 12.9	409.5 ± 37.5^b^	5.6 ± 0.8^b^	22.6 ± 2.3^a^
OO-L	152.8 ± 16.8	474.7± 22.5^b^	7.1 ± 0.6^b^	22.8 ± 1.6^a^
OO-H	165.0 ± 17. 4	469. 4±21.3^b^	6.7 ± 0.3^b^	24. 2 ± 3.6^a^

^a^Compared with NC, *P* < 0.05; ^b^compared with HFD-B, *P* < 0.05

### Ratio of liver and kidney weights to body weight

The ratios of the liver and kidney weights to body weight were expressed as relative weight per 100 g body weight (Table 3). The ratio of liver to body weight was higher in the HFD-A and HFD-B groups than in the NC group, while the ratio of kidney weight to body weight was the opposite. Additionally, the ratios of liver to body weight in the GO-M, GO-H, OO-L and OO-H groups and the ratios of kidney weight to body weight in the GO-M, OO-L, OO-H groups were significantly higher than that in the HFD-B group.

**Table 3:** The ratio of liver and kidney weights to body weight at the end of the experiment ($\overline{x}\pm s$, *n* = 12)

Group	ratio of liver to body weight (g/100 g)	ratio of kidney to body weight (g/100 g)
NC	3. 34 ± 0.45	0.90 ± 0. 18
HFD-A	4.86 ± 0.42^a^	0.67 ± 0.10^a^
HFD-B	4.85 ± 0.32^a^	0.65 ± 0.07^a^
GO-L	5. 11±1.71	0.75 ± 0.26
GO-M	5.37 ± 0.58 ^b^	0.85 ± 0.21^b^
GO-H	6.07 ± 0. 22^b^	0.81 ± 0.27
OO-L	5.60 ± 0.81^b^	0.78 ± 0. 14^b^
OO-H	5.94 ± 0.81^b^	0.82 ± 0. 17^b^

^a^Compared with NC, *P* < 0.05; ^b^compared with HFD-B, *P* < 0.05

### Lee index and ratio of adipose tissues to body weight

The Lee index can reflect the level of body fat in an animal. All of the rats were analyzed, and the results indicated a significant increase in adipose tissue in the HFD-A and HFD-B groups. The Lee index of the rats in the HFD groups was significantly higher than that in the other groups, as shown in Table 4. Additionally, the Lee index in the GO-M, GO-H, OO-L and OO-H groups was significantly lower than that in the HFD-B group. Additionally, all of the epididymal fat pads and perirenal fat pads were dissected out and weighed; the actual and relative weights in the HFD-A and HFD-B groups were greater than those in the NC group. Compared with the HFD-B group, the epididymal fat pads in the GO-M, GO-H, OO-L and OO-H groups and the perirenal fat pads in the GO-H and OO-H were significantly lower in weight, and the ratios of adipose tissue to body weight in the GO-H, OO-L and OO-H groups were also significantly lower than those in the HFD-B group.

**Table 4:** Fat pad weight, the ratio of adipose tissue to body weight and Lee’s index in different groups at the end of the experiment ($\overline{x}\pm s$, *n* = 12)

Group	Epididymal fat pad (g)	Perirenal fat pad (g)	Ratio of adipose tissue to body weight (g/100 g)	Lee index
NC	8. 22 ± 0.53	8.48 ± 0.25	3.73 ± 0.30	296.9 ± 9.8
HFD-A	10.43± 2.10^a^	12.75± 2.15^a^	4.29 ± 0.70^a^	307.6± 11.1^a^
HFD-B	10.49 ± 1.53^a^	12.58 ± 3.35^a^	4.30 ± 0.63^a^	310. 2 ± 7.9^a^
GO-L	9.51 ± 3.18	11.88 ± 4.75	4.48 ± 1.26	305.6 ± 11.9
GO-M	7.14 ± 1.68^b^	10.01 ± 3.09	3.80 ± 0.89	302.3 ± 7.7^b^
GO-H	6.42 ± 1.58^b^	6.65 ± 1.86^b^	3.18 ± 0.53^b^	301.4 ± 13.3^b^
OO-L	7.07 ± 2.08^b^	9.99 ± 3.02	3.59 ± 0.61^b^	301.4 ± 8.7^b^
OO-H	8.49 ± 0.99^b^	9.38± 2.35^b^	3.80 ± 0.52^b^	296.4 ± 6.6^b^

^a^Compared with NC, *P* < 0.05; ^b^compared with HFD-B, *P* < 0.05

### Serum lipid concentrations

Table 5 includes the serum levels of TG and TC for the rats in the different groups at the beginning of the experiment, indicating that there were no significant differences among the eight groups at that time.

**Table 5:** Serum levels of triglycerides (TG) and total cholesterol (TC) in rats fed the basic diet and high-fat diet at beginning of the experiment (mmol/L, $\overline{x}\pm s$, *n* = 12)

Group	TG	TC
NC	1.75 ± 0.84	1.95 ± 0.68
HFD-A	1.96 ± 0.65	2.11 ± 0.24
HFD-B	1.79 ± 0.82	2.16 ± 0.24
GO-L	1.85 ± 0.51	2.22 ± 0.27
GO-M	1.61 ± 0.75	2.15 ± 0.34
GO-H	1.40 ± 0.75	1.98 ± 0.33
OO-L	1.60 ± 0.77	2.14 ± 0.23
OO-H	1.77 ± 0.79	2.04 ± 0.21

At the end of the experiment, the serum levels of TG, TC, and LDL-C in the HFD-A and HFD-B group rats were significantly higher than those in the NC group rats, while HDL-C was significantly lower, which prompted the successful establishment of the hyperlipidemia model. The results listed in Table 6 further illustrate that garlic oil, particularly in the high dose group, had the ability to decrease the level of TC while increasing the level of HDL-C compared with the rats in the HFD-B group. Additionally, onion oil, especially in the high dose group, had the ability to decrease the levels of TG, TC and LDL-C in the serum while increasing the HDL-C level at same time compared with the rats in the HFD-B group.

**Table 6:** Serum levels of TG, TC HDL-C and LDL-C in rats fed the basic diet and high-fat diet at the end of the experiment (mmol/L, $\overline{x}\pm s$, *n* = 12)

Group	TG	TC	HDL-C	LDL-C	Atherosclerosis index
NC	0.96 ± 0.19	1.65 ± 0.29	0.81 ± 0.19	0.58 ± 0.24	1.12 ± 0.60
HFD-A	1.71 ± 0.60^a^	3.11 ± 0.52^a^	0.32 ± 0.18^a^	1.68 ± 0.39^a^	6.33 ± 2.02^a^
HFD-B	1.88 ± 0.71^a^	3.42 ± 0.52	0.31 ± 0.13^a^	1.65 ± 0.32	7.86 ± 1.46^a^
GO-L	1.52 ± 0.29	2.80 ± 0.45	0.55 ± 0.33^b^	1.57 ± 0.34	3.67± 2.00^b^
GO-M	1.75 ± 0.31	2.54 ± 0.56^b^	0.56 ± 0.35^b^	1.54 ± 0.40	4.54 ± 2.11^b^
GO-H	1.49 ± 0.32	2.12 ± 0.58^b^	0.62 ± 0.19^b^	1.51 ± 0.48	2.54 ± 1.01^b^
OO-L	1.33 ± 0.31^b^	2.90 ± 0.40^b^	0.54 ± 0.15^b^	1.50 ± 0.27	4.18 ± 2.01^b^
OO-H	1.22 ± 0.23^b^	2.74 ± 0.31^b^	0.63 ± 0.22^b^	1.21 ± 0.23^b^	4.56 ± 2.04^b^

^a^Compared with NC, *P* < 0.05; ^b^compared with HFD-B, *P* < 0.05

Compared with the rats in the HFD-B group, the atherosclerosis indices of the rats in the garlic oil and onion oil groups were significantly lower, while they were higher in the HFD-A and HFD-B groups than in the NC group (*P* < 0.05), as shown in Table 6.

### The pathological changes in the liver

**Fig. 1:**
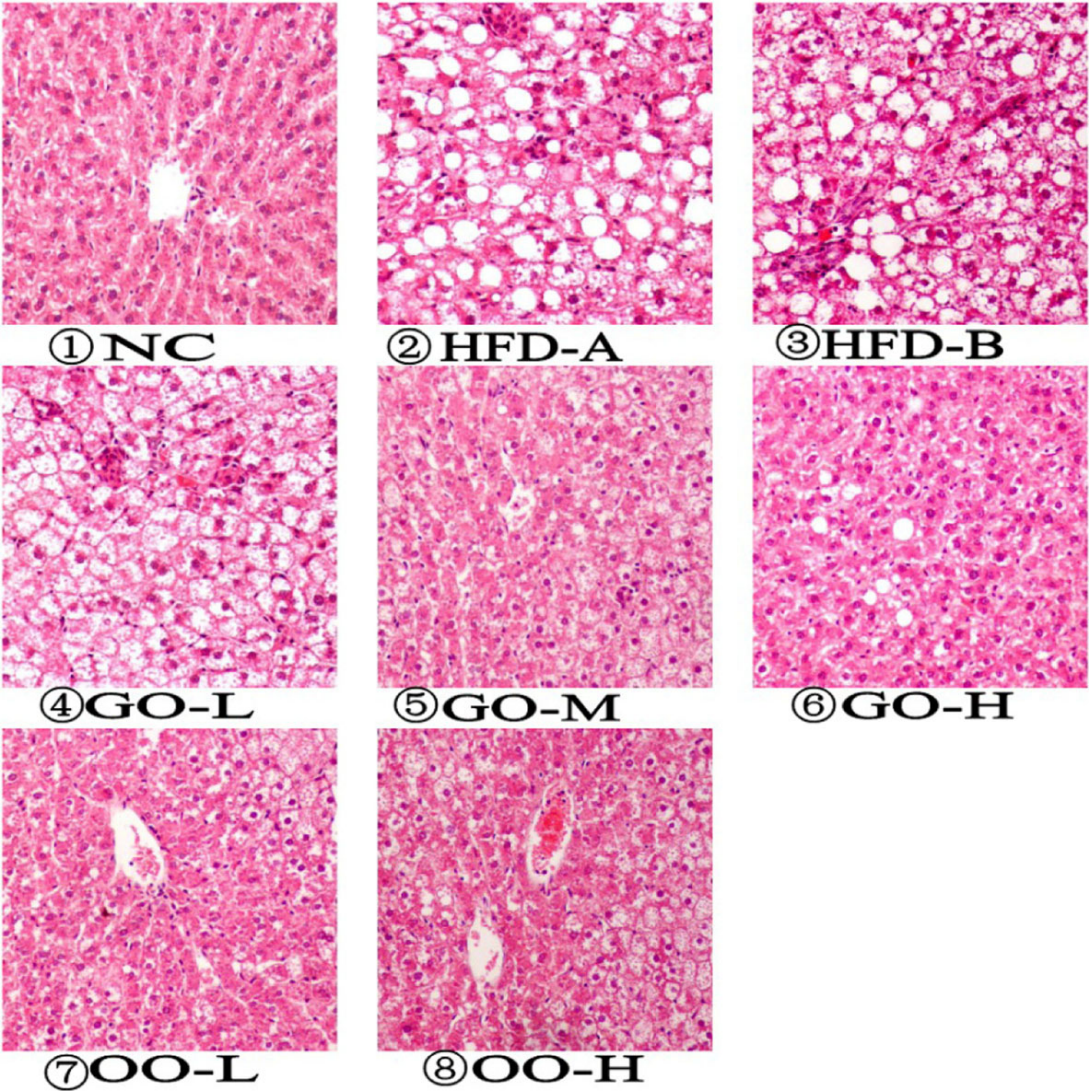
Effects of garlic essential oil and onion essential oil on the liver shown by histological analysis of livers from rats in different groups (liver sections were stained with hematoxylin and eosin; magnification_400)

Table 7 includes the results of the histopathological examination of the livers. The results indicated that the livers of rats fed a high-fat diet were severely degenerated compared with the rats in the NC group. Liver histopathological examinations showed that there were different extents of cell steatosis in the hepatic lobules in rats from the groups fed a high-fat diet. In the NC group rats, there were no lipid droplets distributed in the hepatic lobule, and no cell steatosis or necrosis was found, meaning that the integrity of the lobular structure was maintained. The rats in the HFD-A and HFD-B groups, however, suffered from cell steatosis of the hepatic lobule, cell puffing, and inflammatory cell infiltration in the hepatic lobule and portal area, as shown in Fig. 1. It could therefore be concluded from Table 7 and Fig. 1 that the garlic oil and onion oil alleviated the hepatic steatosis of the hyperlipidemia model in rats.

**Table 7:** The histopathologic observations of the liver of rats under a microscope (HE staining)

Group	N	Numbers for different situations of steatosis			
		Null^a^	Little^b^	Middle^c^	Serious^d^
HFD-A	11	0	1	2	8
HFD-B	11	0	1	1	9
GO-L	10	0	1	3	6
GO-M	12	0	3	4	5
GO-H	11	0	5	4	2
OO-L	12	0	3	4	5
OO-H	12	0	6	4	2

^a^“Null” means that the liver has no apparent disease

^b^“Little” means that cell steatosis of the hepatic lobule is within 25%

^c^“Middle” means that cell steatosis of the hepatic lobule is within 50%

^d^“Serious” means that cell steatosis of the hepatic lobule is greater than 50%

## Discussion

Dyslipidemia and obesity have been gaining significant attention from public health officials in developing countries because they can result in an astonishing increase in the risk factors of diabetes, hepatic adipose infiltration, and cardiovascular disease. In view of the perceived role of food and nutrition in the etiology of chronic diseases and in their prevention, effective nutritional intervention strategies for preventing or managing chronic diseases would be desirable. In this context, it would be most appropriate to evaluate the nutraceutical potential of non-nutrient phytochemicals, especially phytochemicals with hypolipidemic and antioxidant properties.

Obesity is a cardiometabolic risk factor that adversely affects serum lipids by increasing TG levels and decreasing HDL-C levels [26]. The consumption of a high-fat diet, which could facilitate the development of a positive energy balance and lead to an increase in visceral fat deposition, is considered a major risk factor for obesity [26, 27]. It has been reported that some components in garlic and onion are known to be effective in the treatment of obesity and hyperlipidemia [28–30].

This animal study has evaluated the anti-obesity and hypolipidemic effects of onion oil and garlic oil on hyperlipidemia experimentally induced through the feeding of a high-fat diet. The results revealed that the body weight gains of the rats in the HFD groups were significantly higher than that in the NC group, and the weight gains in the garlic oil and onion oil groups were normalized to or lower than that in the NC group (Table 2). In particular, the epididymal fat pad weight, the perirenal fat pad weight, the ratio of adipose tissue weights to body weight, the ratio of liver weight to body weight and the Lee index were higher in the HFD-B group rats than in the NC group (Table 3). In the present study, the anti-obesity activity of the onion oil and garlic oil was examined in rats fed an HFD by measuring the Lee index and markers of obesity. The epididymal fat pad weight, the perirenal fat pad weight, the ratio of adipose tissue weight to body weight, and the Lee index were found to be significantly lower in the GO and OO group rats than in the HFD-B group. These results suggest that garlic oil and onion oil have anti-obesity abilities. It has previously been suggested that onion or garlic extract may provide beneficial anti-obesity effects [29, 31, 32]. In this study, the ratio of liver weight to body weight and the ratio of kidney weight to body weight in some intervention groups were significantly higher than those in the HFD-B group. These results may suggest that garlic oil and onion oil result in adverse effects on the liver, although the organ coefficient has its limitations [33]. The individual differences between animals and their source, initial weight, gender, batches, feeding season and environmental conditions all affect the normal range of growth and development and organ index [34].

Coronary heart disease risk is positively associated with TG, TC and LDL-C and is inversely associated with HDL-C. High levels of TC, TG and LDL-C lead to a high risk for the development of coronary heart disease and atherosclerosis in middle-aged adults [25]. Our data demonstrated that garlic oil and onion oil improved the lipid profile by lowering serum TC, TG, and LDL-C concentrations and the atherogenic index compared with the HFD-B group. As shown in Table 6, the serum TC concentration in the rats in the HFD-A (3.11 mmol/L) group increased by 88.9% compared with the NC group (1.65 mmol/L). However, the serum TC concentrations of the rats in the GO-H and OO-H groups decreased by 38.1 and 19.6%, respectively, compared with the HFD-B group. Similar results have been documented in the reports of Bordia and others [35]. As shown in Table 6, the serum TG concentration (1.71 mmol/L) in the rats in the HFD-A group increased by 76.8% compared with the NC group (0.96 mmol/L). However, the serum TG concentrations of the rats in the GO-H and OO-H groups decreased by 20.6% and 35.1%, respectively, compared with the HFD-B group. The significant reduction in TC and TG with garlic oil and onion oil administration suggests potential health benefits through reductions in obesity, hyperlipidemia and/or similar to those described in the field of malignant cholesterol diseases. Moreover, the HDL-C concentration in the garlic oil and onion oil groups significantly increased compared with the HFD-B group (Table 6). Our findings are in agreement with other studies that have shown that garlic and onion consumption increased HDL-C levels in hyperlipidemic patients [36], animals[37], and patients with coronary artery disease [38]. Finally, our results clearly demonstrated that the contributing dosages of garlic oil and onion oil were 46.3 mg/kg•bw/d (GO-M, OO-L) and 92.6 mg/kg•bw/d (GO-H, OO-H), rather than 11.6 mg/kg•bw/d (GO-L) (Tables 2, 3, 4 and 6). That is, the lower garlic dose had no obvious anti-obesity or hypolipidemic effects.

The doses of garlic oil and onion oil used in our study were sufficient to obtain the physiological benefits without any side effects. As shown in Fig. 1, histological liver analyses revealed that the rats fed an HFD had severe infiltrations of inflammatory cells (Fig. 1 ② and ③), whereas the basal diet-fed rats exhibited normal and healthy histochemical structures (Fig. 1 ①). Additionally, the rats in the garlic oil and onion oil groups had greatly reduced infiltrations of inflammatory cells (from ④ to ⑧ in Fig. 1) compared with the HFD group. In conclusion, garlic oil and onion oil could play important roles in altering body fat and regulating lipid metabolism.

The present study showed that the anti-obesity and hypolipidemic effects of onion oil and garlic oil in the rodent may be beneficial for human health, as onions and garlic are sustainable resources, and many components in their volatile oils have high application value. In addition to their traditional value as foodstuffs, they can also be widely applied to medicine, food additives, feed and other fields with broad prospects for development [39, 40].

## Conclusion

In conclusion, this study may have important implications because it is the first report demonstrating that garlic oil and onion oil have anti-obesity effects and improve the lipid profile in high-fat diet fed rats. However, further study is needed to investigate which compounds in garlic oil and onion oil are responsible for the effects, as well as to determine the molecular mechanisms responsible for the anti-obesity and hypolipidemic activities.

## Additional files

Additional file 1: **Table S1.** The raw data on body weight evolution which is a supplement to Table 2. (XLSX 19 kb)

Additional file 2: **Table S2.** The raw data on food intake which is a supplement to Table 2. (XLS 89 kb)

## Abbreviations

ACSOs: ALK(EN)-based cysteine sulfoxides
HDL-C: High-density Lipoprotein Cholesterol
LDL-C: Low-density Lipoprotein Cholesterol
TC: Total Cholesterol
TG: Triglyceride
